# Limitations of Murine Models for Assessment of Antibody-Mediated Therapies or Vaccine Candidates against Staphylococcus epidermidis Bloodstream Infection

**DOI:** 10.1128/IAI.01472-15

**Published:** 2016-03-24

**Authors:** Leah E. Cole, Jinrong Zhang, Augustus Kesselly, Natalie G. Anosova, Hubert Lam, Harry Kleanthous, Jeremy A. Yethon

**Affiliations:** Sanofi Pasteur, Cambridge, Massachusetts, USA

## Abstract

Staphylococcus epidermidis is normally a commensal colonizer of human skin and mucus membranes, but, due to its ability to form biofilms on indwelling medical devices, it has emerged as a leading cause of nosocomial infections. Bacteremia or bloodstream infection is a frequent and costly complication resulting from biofilm fouling of medical devices. Our goal was to develop a murine model of S. epidermidis infection to identify potential vaccine targets for the prevention of S. epidermidis bacteremia. However, assessing the contribution of adaptive immunity to protection against S. epidermidis challenge was complicated by a highly efficacious innate immune response in mice. Naive mice rapidly cleared S. epidermidis infections from blood and solid organs, even when the animals were immunocompromised. Cyclophosphamide-mediated leukopenia reduced the size of the bacterial challenge dose required to cause lethality but did not impair clearance after a nonlethal challenge. Nonspecific innate immune stimulation, such as treatment with a Toll-like receptor 4 (TLR4) agonist, enhanced bacterial clearance. TLR2 signaling was confirmed to accelerate the clearance of S. epidermidis bacteremia, but TLR2^−/−^ mice could still resolve a bloodstream infection. Furthermore, TLR2 signaling played no role in the clearance of bacteria from the spleen. In conclusion, these data suggest that S. epidermidis bloodstream infection is cleared in a highly efficient manner that is mediated by both TLR2-dependent and -independent innate immune mechanisms. The inability to establish a persistent infection in mice, even in immunocompromised animals, rendered these murine models unsuitable for meaningful assessment of antibody-mediated therapies or vaccine candidates.

## INTRODUCTION

Among the coagulase-negative staphylococci, Staphylococcus epidermidis is a ubiquitous colonizer of human skin and mucus membranes and appears to play a vital role in the maintenance of healthy pathogen-free skin (1–4). While S. epidermidis is generally harmless, it can act as an opportunistic pathogen when it breaches the epithelial barrier in individuals with indwelling medical devices such as central venous catheters, ventricular shunts, artificial heart valves, or prosthetic joints. These implanted devices can become coated with S. epidermidis biofilms (reviewed in references 5 and 6), enabling them to act as a nidus for secondary infections such as bacteremia and even septicemia. Immunocompromised or immunosuppressed individuals (7, 8) as well as preterm neonates (9–11) are particularly susceptible to these secondary infections. As S. epidermidis infections are primarily acquired during hospitalization, they are increasingly resistant to antimicrobial drugs (12). Approximately 70% of clinical S. epidermidis isolates have acquired methicillin resistance, and many carry additional resistance to other antimicrobial classes (13, 14). Furthermore, the bacterial isolates responsible for these infections are far more likely to possess the *ica* operon carrying genes for biofilm formation than their commensal counterparts found in the community (15). Therefore, due to increased antibiotic resistance and the natural recalcitrance of these biofilms with respect to antimicrobials, surgical removal of the device is often the only effective treatment option for implant-associated S. epidermidis infection.

Approximately 1 of every 25 inpatients in U.S. acute care hospitals experiences a health care-associated infection (16), and data from the National Healthcare Safety Network show that coagulase-negative staphylococci are a leading cause (17). It has been estimated that ∼41,000 cases of central line-associated bloodstream infection (CLABSI) occurred in U.S. hospitals in 2009, with a similar number occurring in outpatient hemodialysis centers (∼37,000 cases in 2008) (18). Coagulase-negative staphylococci are the leading cause of CLABSI (20.5%) (17), and S. epidermidis accounts for >70% of catheter-related infections within that group (19, 20). CLASBSI has been shown to increase hospital costs as well as length of stay (21–23), and direct medical costs are approximately $20,000 per occurrence (24, 25). S. epidermidis infections are particularly severe for very-low-birth-weight neonates, for whom the bacterium is a significant cause of both morbidity and mortality (26). It has been shown that 15% to 27.6% of very-low-birth-weight neonates develop late-onset sepsis and that coagulase-negative staphylococci are responsible for 15% to 64.4% of these infections (27–29). The implementation of bundled intervention programs and other best practices for infection control in the insertion and maintenance of central lines (30, 31) has reduced rates of CLABSI in hospital intensive care units (18). The National Healthcare Safety Network reported a 46% drop in CLABSI rates from 2008 to 2013. However, the ongoing morbidity of S. epidermidis infections begs for additional solutions. To this end, development of an efficacious vaccine or antibody therapy to prevent or eliminate S. epidermidis bloodstream infections would have a significant and beneficial impact on public health.

An important step in vaccine development is the establishment of relevant models to screen and prioritize candidate antigens. Our work focused on the development of a murine model of S. epidermidis bloodstream infection with the ultimate goal of identifying candidate vaccine antigens capable of enhancing the clearance of bacteremia. These efforts revealed two important findings about the role of innate immunity in clearance of S. epidermidis from the blood: (i) both TLR2-dependent and -independent pathways contribute to this process, and (ii) nonspecific innate immune stimulation alone is able to enhance bacterial clearance. Further, we demonstrate an important limitation of using mice to model S. epidermidis bacteremia: challenge either leads to rapid clearance or overwhelms the system and culminates in mortality within 24 h. There is no middle ground in which challenge leads to illness or a persistent bacterial burden. Cyclophosphamide-induced leukopenia reduces the size of the bacterial challenge required to cause lethality but does not impact clearance after a nonlethal challenge. Taken together, these data show that S. epidermidis infection in the mouse is handled efficiently by innate immunity, which limits the utility of murine models for studies of vaccine or antibody efficacy against this opportunistic pathogen.

## MATERIALS AND METHODS

### Bacterial strains, media, and growth conditions.

S. epidermidis strain RP62A was originally isolated from a patient with catheter sepsis, and we obtained it from the ATCC. Its complete genome sequence is available, and its capacity to grow as a biofilm is well documented. For our studies, biofilm-grown bacteria were produced as follows. Two days prior to challenge, 5 ml of tryptic soy broth (TSB) was inoculated with a single bacterial colony of RP62A from an overnight TSB agar plate and then incubated at 37°C with shaking at 250 rpm. After growth for exactly 18 h, 50 μl of this culture was transferred into 5 ml fresh TSB. This culture was grown at 37°C with shaking at 250 rpm for exactly 5 h. This 5-h biofilm starter culture was diluted to an optical density at 600 nm (OD_600_) of 2.0. Next, 2.5 ml of this biofilm starter culture was combined with 22.5 ml of fresh TSB in an empty, sterile 10-cm-diameter petri dish and incubated overnight at 37°C without shaking. After exactly 20 h of growth, bacteria were harvested by gently removing TSB and then scraping the biofilm layer into phosphate-buffered saline (PBS). The challenge mixture was pipetted and sonicated to disperse the bacteria prior to storage on ice until use. The dispersal of biofilm clumps following sonication was confirmed by visual inspection using phase-contrast microscopy. The challenge material was serially diluted in PBS and enumerated on TSB agar to verify the exact challenge dose—this also confirmed that bacteria remained completely viable following sonication. Planktonic bacteria were produced as follows. At 1 day prior to challenge, 5 ml of TSB was inoculated with 8 to 10 individual colonies from a TSB agar overnight plate, and the culture was grown at 37°C with shaking at 250 rpm for exactly 18 h to allow the bacteria to enter the stationary phase. On the day of the challenge, the 18-h culture was diluted 1:100 into fresh TSB and then grown at 37°C with shaking at 250 rpm for 5 h. The volume of the mixture was then adjusted such that the OD_600_ was between 2 and 3 as determined using a standard spectrophotometer with a 1-cm-path-length cuvette. Cells were collected by centrifugation at 8,000 × *g* for 2 min and resuspended in PBS. This planktonic bacterial suspension was maintained on ice prior to use. The bacterial suspension was serially diluted in PBS and grown on TSB agar to verify the exact challenge dose.

### Animals.

Female specific-pathogen-free BALB/c mice (Charles River) as well as C.129(B6)-Tlr2tm1Kir/J (TLR2^−/−^) and C57BL/6J mice (The Jackson Laboratory) were used. All procedures that involved animals were conducted under protocols approved by the Institutional Animal Care and Use Committee and were performed at an AAALAC-accredited facility.

### Cyclophosphamide induction of leukopenia and counting of white blood cells.

Cyclophosphamide monohydrate (Sigma-Aldrich) was dissolved in water to a concentration of 20 mg/ml. Three days prior to challenge, mice were given an intraperitoneal (i.p.) injection at a dosage of 200 mg/kg of body weight. To determine the number of white blood cells present before and after depletion, ACK (ammonium-chloride-potassium) buffer was used to lyse red blood cells in EDTA-treated whole-blood samples. White blood cell counts were performed manually with a hemocytometer.

### Bacteremia challenge model.

Bacterial challenges were administered in a 0.2-ml volume by intravenous (i.v. [tail vein]) or i.p. injection using a 1-ml syringe and 27-gauge 0.5-in. needle. Mice were euthanized at the indicated times, and then blood was collected via cardiac bleed and stored in BD Microtainer MAP Microtubes containing K_2_EDTA to prevent coagulation. Blood was serially diluted in PBS and plated on TSB agar plates with 50 μg/ml kanamycin (TSA-Kan) (Teknova) prior to incubation (37°C, 24 h); S. epidermidis colonies were counted manually, in a blinded fashion. Spleens were harvested at the indicated times and stored on ice in sterile 1.2-ml polypropylene tubes until ready for processing. All tubes were centrifuged for 10 s to pack spleens into the bottom of the tube. Next, approximately 0.3 g of autoclaved zirconium oxide beads (1.0-mm diameter) was loaded on top of the spleens followed by 300 μl sterile PBS (CellGro; Mediatech). Spleens were homogenized using a NextAdvance Bullet Blender Blue tissue homogenizer at speed setting 8 for 3 min. Tubes were visually inspected to ensure complete homogenization. Spleen homogenates were serially diluted and counted as described above. For experiments involving administration of TLR4 agonist, 3 days prior to bacterial challenge, mice were given subcutaneous (s.c.) injections of a 0.2-ml volume in the scapular region containing 2 μg of E6020 (Eisai, Tokyo, Japan) or an equivalent volume of PBS vehicle.

### Statistical analysis.

All statistical analyses were performed using GraphPad Prism version 6.01.

## RESULTS

### Immunocompetent mouse model of S. epidermidis infection.

**FIG 1:**
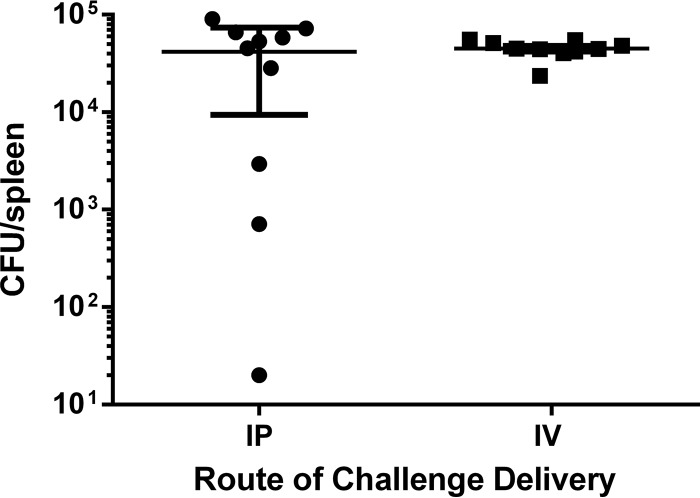
Intravenous delivery produces a more consistent splenic bacterial burden at 24 h post-S. epidermidis challenge. BALB/c WT mice were challenged either i.v. or i.p. with 1.3 × 10^8^ CFU of biofilm-grown S. epidermidis. Splenic bacterial burden was assessed at 24 h postchallenge. Each data point represents a single animal. The bars represent the group means, and the error bars represent the standard deviations.

**FIG 2:**
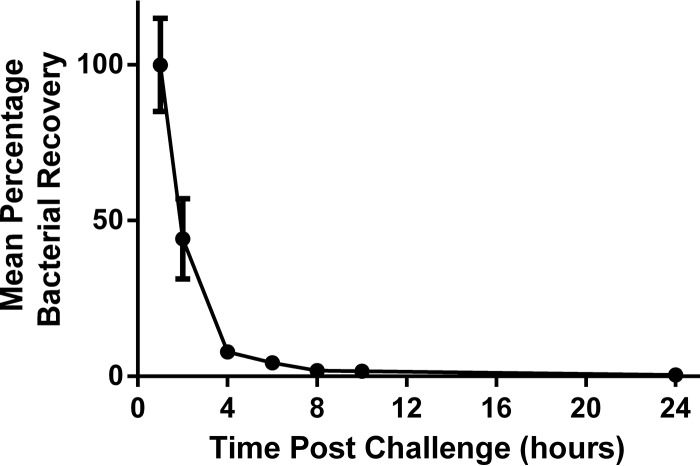
Splenic bacterial burden decreased rapidly after S. epidermidis challenge in immunocompetent mice. Groups of 5 BALB/c WT mice were challenged i.v. with 2.32 × 10^8^ CFU of biofilm-grown S. epidermidis. The bacterial burden was assessed in spleens at the indicated times postchallenge. The percentage of bacterial recovery was calculated by dividing the calculated splenic bacterial burden for an individual animal by the mean splenic bacterial burden at 1 h postchallenge multiplied by 100. Each data point represents the mean percentage of bacterial burden in the group at the indicated time point, and error bars display the standard deviations.

Numerous murine models of S. epidermidis bacteremia have been described (32–37). Experimental variables tested in these models include animal age (pups or adults), route of bacterial challenge (i.v. or i.p.), and readouts (CFU in blood or solid organs). While different groups used different bacterial strains, we performed all studies with S. epidermidis clinical isolate RP62A and chose the model described by Sellman and coworkers as our starting point since it was used to screen and identify potential vaccine candidates (34). In this model, female BALB/c mice were immunized with recombinant S. epidermidis vaccine antigens and challenged via i.p. injection with planktonic S. epidermidis 1 week after the final immunization. Vaccine efficacy was assessed in the spleen and bloodstream after 24 h, since the bacteria were naturally cleared by unimmunized mice at 48 h (34). In an effort to better model the scenario for bloodstream infections arising from biofilms on indwelling devices, we challenged animals with biofilm-grown bacteria. To assess the impact of the route of bacterial delivery, levels of splenic bacterial recovery were compared in naive mice after i.p. and i.v. delivery of biofilm-grown bacteria. While there was no statistically significant difference in the levels of recovery of bacteria from the spleen after 24 h (*P* = 0.7243 [Mann-Whitney]), i.v. delivery led to less variability (Fig. 1) and was therefore selected for subsequent experiments. We performed a longitudinal study on the splenic bacterial burden from 1 to 24 h postchallenge to determine the best time to assess the contribution of antigen-specific antibody to bacterial clearance (Fig. 2). We hoped to identify a window of time during which the bacterial burden was constant but persistent infection was not observed at any time point. Of note, >90% of bacteria in the spleen were eliminated between hours 1 and 4 postchallenge, and this rose to 99.5% elimination by 24 h.

**FIG 3:**
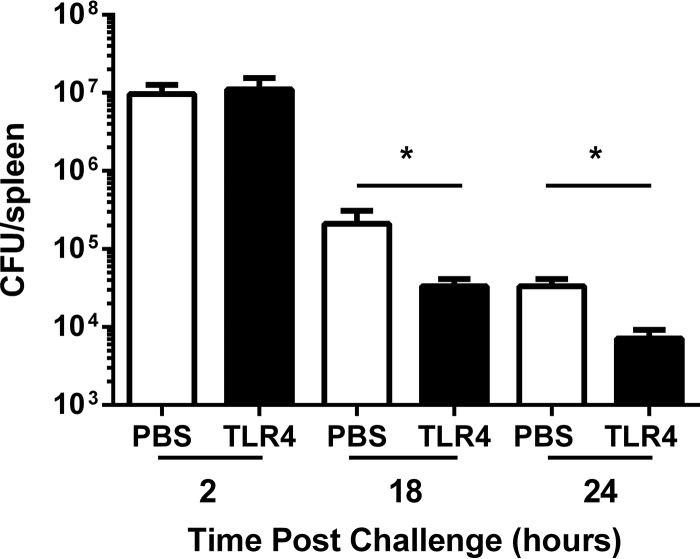
TLR4 stimulation prior to S. epidermidis challenge reduced splenic bacterial burden at 18 and 24 h postchallenge relative to the results seen with PBS-treated control animals. Three days prior to bacterial challenge, groups of 5 immunocompetent BALB/c WT mice were s.c. injected with 200 μl of PBS or a TLR4 agonist (E6020). Mice were challenged i.v. with 1.8 × 10^8^ CFU (2 and 24 h) or 6.56 × 10^8^ CFU (18 h) of biofilm-grown S. epidermidis. The splenic bacterial burden at 2, 18, or 24 h postchallenge is shown. Bars represent the mean bacterial burden for the group, and error bars represent standard deviations. *, statistically significant difference in splenic bacterial burden between PBS-treated and TLR4 agonist-treated mice at 18 h (*P* = 0.0079 [Mann-Whitney]) and 24 h (*P* = 0.0079 [Mann-Whitney]) postchallenge.

Since the innate immune response was very effective at mediating rapid clearance of S. epidermidis, we considered the possibility that any treatment that stimulated innate immunity would enhance bacterial clearance. To test this hypothesis, naive mice were injected 3 days prior to challenge with either PBS or a TLR4 agonist (E6020). A TLR4 agonist was selected because TLR4 signaling has been shown not to be involved in cytokine production induced by S. epidermidis (32); human embryonic kidney cells transfected with TLR2 (but not TLR4/MD-2) dramatically increase interleukin-8 (IL-8) production in response to S. epidermidis, and preincubation of whole human blood with neutralizing anti-TLR2 (but not anti-TLR4) antibodies inhibits S. epidermidis-induced IL-6 production (32). Despite TLR4 having no identified role in S. epidermidis signaling, injection of a TLR4 agonist led to statistically significant 5.8- and 4.9-fold reductions in the splenic bacterial burden at 18 and 24 h, respectively (Fig. 3).

To test whether innate immune stimulation such as might occur during a mouse vaccine study could enhance bacterial clearance, we “immunized” naive mice three times with 10 μg of E. coli lipopolysaccharide (LPS) on days 0, 28, and 35 and then challenged them with S. epidermidis 1 week after the final immunization. E. coli LPS was selected because recombinant protein antigens are often produced in E. coli and may contain residual LPS. This immunization with E. coli LPS alone was able to reduce the bacterial burden of S. epidermidis in the spleen after 24 h (data not shown).

Taken together, these data demonstrate that mice mount a highly effective innate immune response that naturally clears S. epidermidis challenge. These data also suggest that any innate immune stimulation, whether specific to S. epidermidis or not, can enhance S. epidermidis clearance. Therefore, to properly assess the contribution of antigen-specific adaptive immunity, we sought to develop a model in which innate immunity was impaired.

### Leukopenic model of S. epidermidis infection.

**FIG 4:**
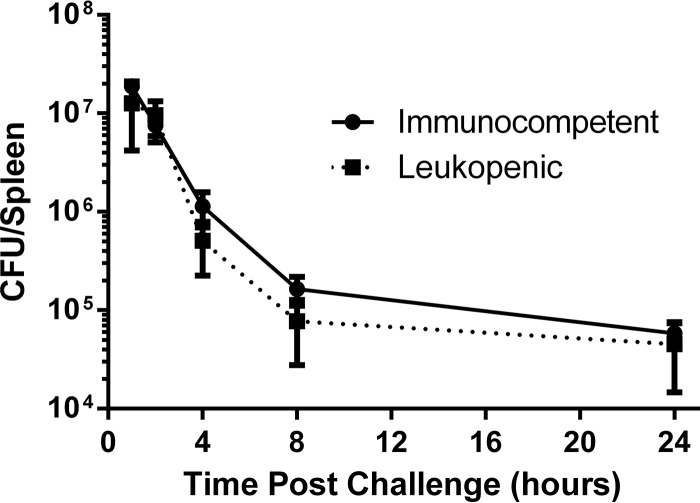
Cyclophosphamide-induced leukopenia does not impair clearance of a nonlethal S. epidermidis challenge. Groups of 5 mice, including either PBS-pretreated immunocompetent mice or cyclophosphamide-pretreated leukopenic mice, were challenged i.v. with 2 × 10^8^ CFU of biofilm-grown S. epidermidis. The bacterial burden was assessed in spleens at the indicated time points. Each data point represents mean splenic bacterial burden in the group at the indicated time point, and error bars display the standard deviations.

Cyclophosphamide-induced neutropenia impairs the ability of mice to control S. epidermidis infection, and challenge with 10^9^ CFU can be lethal within 24 h (33). We hypothesized that challenging leukopenic mice with a lower dose would lead to sustained infection without progression to death, thereby creating an appropriate window of opportunity to evaluate vaccine efficacy. Treatment of mice with 200 mg/kg cyclophosphamide reduced the circulating white blood cell (WBC) counts by >90% on days 3 and 4 postinjection (data not shown). We therefore challenged mice 3 days after treatment so that the entire experiment would fall within the leukopenic window. Cyclophosphamide- or PBS-treated mice were challenged by i.v. injection with 2 × 10^8^ CFU of biofilm-grown bacteria, and the splenic bacterial burden was then assessed from 1 to 24 h postchallenge (Fig. 4). Both leukopenic and immunocompetent animals were able to clear the bacterial challenge, and there was no statistically significant impact of cyclophosphamide treatment on the CFU recovered from spleens (*P* = 0.2936 [two-way analysis of variance {ANOVA}]). As challenge of leukopenic mice with doses of biofilm-grown bacteria equal to or greater than 8 × 10^8^ CFU per 0.2 ml could rapidly lead to a moribund state requiring immediate euthanasia (data not shown), all subsequent experiments were done with only planktonic bacteria.

### Titrating the challenge dose in both immunocompetent and neutropenic mouse models.

Splenic bacterial burden was assessed in both immunocompetent and leukopenic mice after challenge with various doses of planktonic bacteria. The goal was to identify challenge doses that led to persistent yet nonlethal infection. Regardless of the mouse immune status, planktonic bacterial challenge resulted in either rapid clearance or bacterial overgrowth and death (Table 1). For immunocompetent mice, the tipping point was between 1.0 × 10^9^ and 2.3 × 10^9^ CFU. For cyclophosphamide-treated mice, the transition from clearance to overgrowth happened at approximately 6.6 × 10^8^ CFU. Since we showed in Fig. 3 that a TLR4 agonist could enhance bacterial clearance after a nonlethal challenge, we wanted to assess if it would offer protection against an otherwise lethal challenge. However, we saw no protection when either immunocompetent or leukopenic E6020-primed mice were challenged with 2 × 10^9^ CFU of S. epidermidis (data not shown).

**TABLE 1:** Outcomes of challenge of groups of 5 or 6 immunocompetent or leukopenic mice with various doses of planktonic *S. epidermidis*

Immune status	Challenge dose (CFU)	Challenge outcome
Immunocompetent	2.9 × 10^8^	Clearance
	6.6 × 10^8^	Clearance
	1 × 10^9^	Clearance
	2.3 × 10^9^	No clearance
Leukopenic	1.8 × 10^8^	Clearance
	3.5 × 10^8^	Clearance
	6.6 × 10^8^	Clearance (3 animals) and no clearance (2 animals)
	7 × 10^8^	No clearance
	1 × 10^9^	No clearance
	2.3 × 10^9^	No clearance

### TLR2^−/−^ model of S. epidermidis infection.

**FIG 5:**
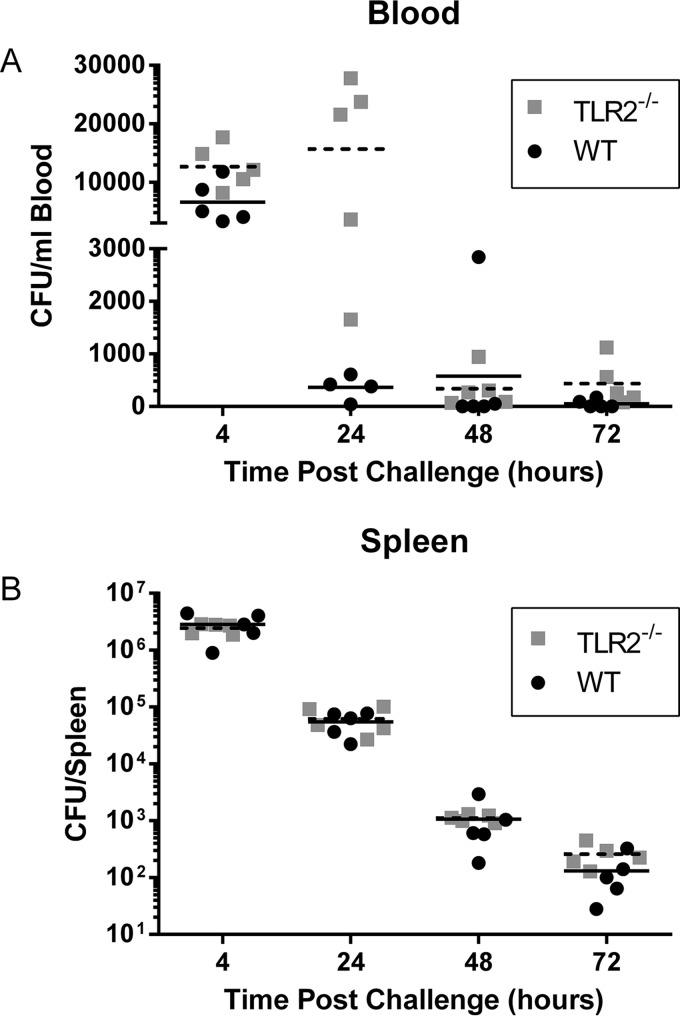
Loss of TLR2 leads to delayed clearance of S. epidermidis from the bloodstream but has no impact on clearance of bacteria from spleen. Groups of 5 animals per time point of WT (black circles) and TLR2^−/−^ (gray squares) mice were challenged i.v. with 2.6 × 10^8^ CFU of S. epidermidis. Bacterial burden was assessed in blood (A) and in spleens (B) at the indicated time points. Each data point represents a single animal. For both panel A and panel B, the solid bar represents the mean of the results from the WT group and the dashed bar represents the mean of the results from the TLR2^−/−^ group.

Since we could not establish persistent infection in leukopenic mice, we assessed the impact of a more targeted impairment of innate immunity. TLR2^−/−^ mice are reported to show impaired clearance of an S. epidermidis challenge, as evidenced by high levels of bacteria in the peripheral blood at 24 and 48 h postchallenge (32). We attempted to confirm and expand on these observations in TLR2^−/−^ mice by evaluating the bacterial burden in both the blood and spleen at 4, 24, 48, and 72 h postchallenge (Fig. 5). Bacteria are rapidly cleared from wild-type (WT) mice as the mean bloodstream bacterial burden drops between 4 and 24 h postchallenge. In agreement with published results (32), the mean bloodstream bacterial burden in TLR2^−/−^ mice did not decrease between 4 and 24 h postchallenge. However, in contrast to previously published studies (32), the bacteria were effectively being cleared by 48 h in the TLR2^−/−^ mice. There was not even a transient increase in bacterial burden in the spleens of TLR2^−/−^ mice as the splenic bacterial burden decreased over all the time points examined, and there was no statistical difference in the splenic bacterial counts between the WT and TLR2^−/−^ mice at any of the time points examined (*P* = 0.5785 [two-way ANOVA]). These data suggested that while TLR2 may be involved in the clearance of S. epidermidis bacteremia, other innate immune pathways are also at play.

**FIG 6:**
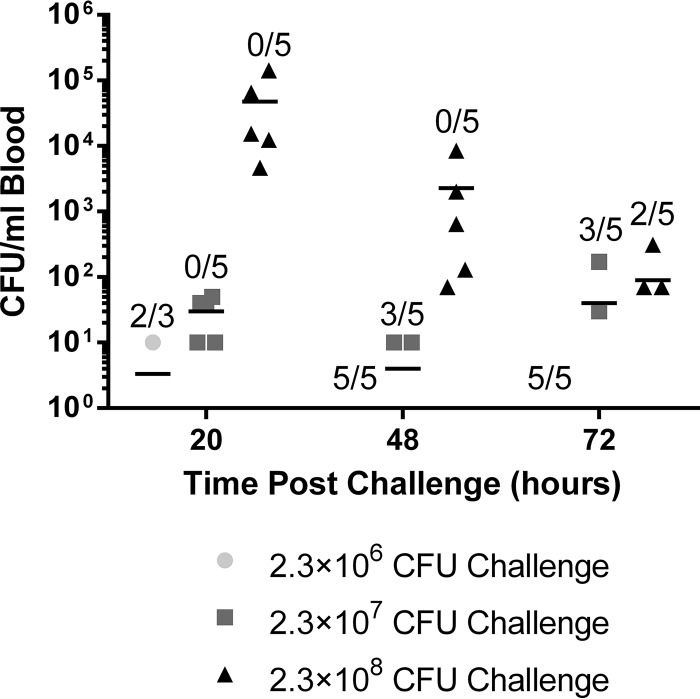
Reduction in the S. epidermidis challenge dose accelerated the clearance of bacteria from the bloodstream of TLR2^−/−^ mice. Groups of 3 to 5 TLR2^−/−^ mice were challenged i.v. with 2.34 × 10^6^, 2.34 × 10^7^, or 2.34 × 10^8^ CFU. The bacterial burden was assessed in blood at the indicated time points. Data points indicate the levels of CFU per milliliter for individual animals. Each line indicates the mean for each group. Because the *y* axis is logarithmic, only values greater than zero are plotted. The value was zero for 20 mice; therefore, the data for those mice are not visible on the graph. In the displayed fractions, the numerator indicates the number of mice in that group in which 0 CFU of S. epidermidis/ml was detected in the blood at the specified time point whereas the denominator indicates the total number of mice in that group.

The transient increase that we observed in bloodstream bacterial burden at 24 h followed by a rapid decrease at 48 h led us to ask whether non-TLR2-dependent clearance processes were triggered only upon reaching a threshold concentration of bacteria. If this was the case, challenging animals with a lower dose of S. epidermidis might postpone clearance by delaying the onset of the non-TLR2-mediated clearance mechanisms. To test this hypothesis, we challenged TLR2^−/−^ mice with 2.3 × 10^6^, 2.3 × 10^7^, or 2.3 × 10^8^ CFU per mouse, and the bloodstream burden was assessed at 20, 48, and 72 h postchallenge (Fig. 6). Bacteria were cleared from the bloodstream at all doses tested, suggesting that any non-TLR2-mediated clearance mechanism that was present was still active at lower concentrations of bacteria.

## DISCUSSION

S. epidermidis is a ubiquitous commensal that is part of a healthy skin flora, but if this bacterium breaches the epithelial barrier it can act as an opportunistic pathogen in preterm infants, immunocompromised individuals, and those with indwelling medical devices (reviewed in references 26 and 38). Our goal was to develop a murine model of S. epidermidis infection to test the efficacy of candidate vaccines against S. epidermidis bacteremia. Unfortunately, developing a model of persistent S. epidermidis bloodstream infection proved challenging. For both immunocompetent and leukopenic mice, S. epidermidis challenge with clinical isolate RP62A led to one of two disparate outcomes: either the infection was very rapidly cleared or there was uncontrolled bacterial growth that culminated in death within 24 h. This problem could not be overcome by optimizing the challenge dose. For immunocompetent mice, the tipping point was somewhere between 1.0 × 10^9^ and 2.3 × 10^9^ CFU for a challenge with planktonic bacteria. Cyclophosphamide-induced leukopenia reduced the overall number of bacteria required to overwhelm the immune system to approximately 6.6 × 10^8^ CFU but still did not lead to persistent infection. The ability of cyclophosphamide-treated leukopenic animals to clear infection raises questions about the specific immune cells mediating bacterial clearance. Cyclophosphamide is a chemotherapeutic and immunosuppressant, alkylating, cytotoxic drug and is most toxic to rapidly proliferating cells and tissues. While some immune cells, such as neutrophils, are more susceptible than others, studies have demonstrated broad-based effects on multiple white blood cells in mice (33, 39). In the future, characterization of the immune cell population remaining after cyclophosphamide treatment, combined with bacterial challenge studies in mice depleted of specific immune cell populations, could provide additional insight into the mechanism of innate clearance of S. epidermidis infection.

The absence of a middle ground in which challenge leads to a persistent bacterial burden is problematic, since neither rapid clearance nor rapid death resembles the chronic human infection we are attempting to model. Using a nonlethal S. epidermidis challenge, we would be assessing the acceleration of an ongoing efficacious innate immune system-mediated process. Therefore, any attempt to differentiate vaccine candidates is limited by the noise imposed by innate clearance mechanisms. However, a lethal challenge model is also untenable because of the tipping point phenomenon described above. Schaeffer et al. (40) recently showed that cyclophosphamide immunosuppression alone was not sufficient to establish S. epidermidis bloodstream infection in rats. However, they were successful in establishing infection when the animals were further weakened by implantation of a foreign body (catheter). Unfortunately, their “two-hit” approach to rendering animals susceptible is highly challenging from a technical perspective, and it is unclear whether infection persists or is continually reseeded from a protected biofilm nidus on the implant.

TLR2 has previously been shown to mediate recognition of live S. epidermidis and clearance of bacteremia (32). We found that the loss of TLR2 had no impact on bacterial clearance from the spleen and led to only a transient delay in bloodstream clearance at 24 h postchallenge (Fig. 6). Unfortunately, such a small window of increased susceptibility and the noted variability at that time point rendered TLR2^−/−^ mice an unsuitable model for assessing vaccine candidate efficacy. Our findings with respect to bloodstream clearance of S. epidermidis are in agreement with those of Bi and coworkers (36) but differ from those reported by Strunk et al. (32). All these data suggest that TLR2 accelerates the clearance of bacteremia; however, our data and the work of Bi and coworkers (36) suggest that TLR2 is not necessary for the clearance of bloodstream bacteria. Therefore, while it is clear that TLR2 does play a role in the recognition and clearance of S. epidermidis from the blood, our data demonstrate that additional innate immune pathways are also involved. Indeed, the involvement of other innate signaling pathways in the recognition of S. epidermidis has been described. For example, the presence of S. epidermidis peptidoglycan has been shown to lead to activation of monocyte-like THP-1 cells by both TLR2 and NOD2 (41). TNF production by murine peritoneal macrophages in response to high concentrations of S. epidermidis has been shown to be primarily TLR2 independent (32), and production of IL-17A by live CD45^+^ skin cells has been shown to be independent of TLR2 (4). Therefore, multiple lines of inquiry suggest that various innate immune signaling pathways function in the recognition and elimination of S. epidermidis.

As part of our model optimization efforts, we assessed the ability of potential vaccine antigens to reduce splenic bacterial burden at 24 h postchallenge. Immunization with either SERP0207 (SdrG) (42) or SERP0237 (lipoate-protein ligase A family protein) led to a consistent statistically significant reduction in splenic bacterial burden at 24 h, but our initial preparations of these antigens also led to reactogenicity that manifested as injection site lumps and liver lesions. Further purification yielded protein preparations that were no longer reactogenic, but they also lost the efficacy observed with the earlier batches (unpublished data). Since the impact of TLR4 agonists on S. epidermidis clearance when administered either prior to challenge or on a standard immunization schedule has been observed, it is possible that innate immune system-stimulating contaminants in our exploratory vaccine preparation caused misleading results. This highlights the need for highly purified materials and suggests that routine testing for residual LPS would be prudent for such studies.

Relevant and translational infection models are a crucial step in the development of new vaccines. In conclusion, our work presents valuable information about the innate immune clearance mechanisms that control S. epidermidis bloodstream infections and demonstrates that these innate immunocompetent and immunocompromised mouse models appear to be unsuitable tools for the assessment of candidate vaccines against S. epidermidis bacteremia.

## References

[1] Cogen AL, Yamasaki K, Muto J, Sanchez KM, Crotty Alexander L, Tanios J, Lai Y, Kim JE, Nizet V, Gallo RL 2010 Staphylococcus epidermidis antimicrobial delta-toxin (phenol-soluble modulin-gamma) cooperates with host antimicrobial peptides to kill group A Streptococcus. PLoS One 5:e8557. doi:10.1371/journal.pone.0008557. 20052280 PMC2796718

[2] Lai Y, Cogen AL, Radek KA, Park HJ, Macleod DT, Leichtle A, Ryan AF, Di Nardo A, Gallo RL 2010 Activation of TLR2 by a small molecule produced by Staphylococcus epidermidis increases antimicrobial defense against bacterial skin infections. J Invest Dermatol 130:2211–2221. doi:10.1038/jid.2010.123. 20463690 PMC2922455

[3] Cogen AL, Yamasaki K, Sanchez KM, Dorschner RA, Lai Y, MacLeod DT, Torpey JW, Otto M, Nizet V, Kim JE, Gallo RL 2010 Selective antimicrobial action is provided by phenol-soluble modulins derived from Staphylococcus epidermidis, a normal resident of the skin. J Invest Dermatol 130:192–200. doi:10.1038/jid.2009.243. 19710683 PMC2796468

[4] Naik S, Bouladoux N, Wilhelm C, Molloy MJ, Salcedo R, Kastenmuller W, Deming C, Quinones M, Koo L, Conlan S, Spencer S, Hall JA, Dzutsev A, Kong H, Campbell DJ, Trinchieri G, Segre JA, Belkaid Y 2012 Compartmentalized control of skin immunity by resident commensals. Science 337:1115–1119. doi:10.1126/science.1225152. 22837383 PMC3513834

[5] Huebner J, Goldmann DA 1999 Coagulase-negative staphylococci: role as pathogens. Annu Rev Med 50:223–236. doi:10.1146/annurev.med.50.1.223. 10073274

[6] Van Mellaert L, Shahrooei M, Hofmans D, Eldere JV 2012 Immunoprophylaxis and immunotherapy of Staphylococcus epidermidis infections: challenges and prospects. Expert Rev Vaccines 11:319–334. doi:10.1586/erv.11.190. 22380824

[7] Bearman GM, Wenzel RP 2005 Bacteremias: a leading cause of death. Arch Med Res 36:646–659. doi:10.1016/j.arcmed.2005.02.005. 16216646

[8] Bender JW, Hughes WT 1980 Fatal Staphylococcus epidermidis sepsis following bone marrow transplantation. Johns Hopkins Med J 146:13–15. 6986499

[9] Vergnano S, Menson E, Kennea N, Embleton N, Russell AB, Watts T, Robinson MJ, Collinson A, Heath PT 2011 Neonatal infections in England: the NeonIN surveillance network. Arch Dis Child Fetal Neonatal Ed 96:F9–F14. doi:10.1136/adc.2009.178798. 20876594

[10] Carrieri MP, Stolfi I, Moro ML 2003 Intercenter variability and time of onset: two crucial issues in the analysis of risk factors for nosocomial sepsis. Pediatr Infect Dis J 22:599–609. 12867834 10.1097/01.inf.0000073205.74257.a5

[11] Donowitz LG, Haley CE, Gregory WW, Wenzel RP 1987 Neonatal intensive care unit bacteremia: emergence of gram-positive bacteria as major pathogens. Am J Infect Control 15:141–147. doi:10.1016/0196-6553(87)90137-4. 3651111

[12] Archer GL, Climo MW 1994 Antimicrobial susceptibility of coagulase-negative staphylococci. Antimicrob Agents Chemother 38:2231–2237. doi:10.1128/AAC.38.10.2231. 7840550 PMC284723

[13] Miragaia M, Thomas JC, Couto I, Enright MC, de Lencastre H 2007 Inferring a population structure for Staphylococcus epidermidis from multilocus sequence typing data. J Bacteriol 189:2540–2552. doi:10.1128/JB.01484-06. 17220222 PMC1899367

[14] McCann MT, Gilmore BF, Gorman SP 2008 Staphylococcus epidermidis device-related infections: pathogenesis and clinical management. J Pharm Pharmacol 60:1551–1571. doi:10.1211/jpp/60.12.0001. 19000360

[15] Cherifi S, Byl B, Deplano A, Nonhoff C, Denis O, Hallin M 2013 Comparative epidemiology of Staphylococcus epidermidis isolates from patients with catheter-related bacteremia and from healthy volunteers. J Clin Microbiol 51:1541–1547. doi:10.1128/JCM.03378-12. 23486718 PMC3647944

[16] Magill SS, Edwards JR, Bamberg W, Beldavs ZG, Dumyati G, Kainer MA, Lynfield R, Maloney M, McAllister-Hollod L, Nadle J, Ray SM, Thompson DL, Wilson LE, Fridkin SK 2014 Multistate point-prevalence survey of health care-associated infections. N Engl J Med 370:1198–1208. doi:10.1056/NEJMoa1306801. 24670166 PMC4648343

[17] Sievert DM, Ricks P, Edwards JR, Schneider A, Patel J, Srinivasan A, Kallen A, Limbago B, Fridkin S 2013 Antimicrobial-resistant pathogens associated with healthcare-associated infections: summary of data reported to the National Healthcare Safety Network at the Centers for Disease Control and Prevention, 2009–2010. Infect Control Hosp Epidemiol 34:1–14. doi:10.1086/668770. 23221186

[18] Centers for Disease Control and Prevention (CDC). 2011 Vital signs: central line-associated blood stream infections—United States, 2001, 2008, and 2009. MMWR Morb Mortal Wkly Rep 60:243–248. 21368740

[19] Aldea-Mansilla C, Garcia de Viedma D, Cercenado E, Martin-Rabadan P, Marin M, Bouza E 2006 Comparison of phenotypic with genotypic procedures for confirmation of coagulase-negative Staphylococcus catheter-related bloodstream infections. J Clin Microbiol 44:3529–3532. doi:10.1128/JCM.00839-06. 17021078 PMC1594756

[20] Haslett TM, Isenberg HD, Hilton E, Tucci V, Kay BG, Vellozzi EM 1988 Microbiology of indwelling central intravascular catheters. J Clin Microbiol 26:696–701. 3366864 10.1128/jcm.26.4.696-701.1988 PMC266415

[21] Dimick JB, Pelz RK, Consunji R, Swoboda SM, Hendrix CW, Lipsett PA 2001 Increased resource use associated with catheter-related bloodstream infection in the surgical intensive care unit. Arch Surg 136:229–234. doi:10.1001/archsurg.136.2.229. 11177147

[22] Warren DK, Quadir WW, Hollenbeak CS, Elward AM, Cox MJ, Fraser VJ 2006 Attributable cost of catheter-associated bloodstream infections among intensive care patients in a nonteaching hospital. Crit Care Med 34:2084–2089. doi:10.1097/01.CCM.0000227648.15804.2D. 16763511

[23] Blot SI, Depuydt P, Annemans L, Benoit D, Hoste E, De Waele JJ, Decruyenaere J, Vogelaers D, Colardyn F, Vandewoude KH 2005 Clinical and economic outcomes in critically ill patients with nosocomial catheter-related bloodstream infections. Clin Infect Dis 41:1591–1598. doi:10.1086/497833. 16267731

[24] Kilgore M, Brossette S 2008 Cost of bloodstream infections. Am J Infect Control 36:S171.e7–S171.e12. doi:10.1016/j.ajic.2008.10.009. 19084149

[25] Boyce JM 2012 Prevention of central line-associated bloodstream infections in hemodialysis patients. Infect Control Hosp Epidemiol 33:936–944. doi:10.1086/667369. 22869269

[26] Dong Y, Speer CP 2014 The role of Staphylococcus epidermidis in neonatal sepsis: guarding angel or pathogenic devil? Int J Med Microbiol 304:513–520. doi:10.1016/j.ijmm.2014.04.013. 24881963

[27] Boghossian NS, Page GP, Bell EF, Stoll BJ, Murray JC, Cotten CM, Shankaran S, Walsh MC, Laptook AR, Newman NS, Hale EC, McDonald SA, Das A, Higgins RD; Eunice Kennedy Shriver National Institute of Child Health and Human Development Neonatal Research Network. 2013 Late-onset sepsis in very low birth weight infants from singleton and multiple-gestation births. J Pediatr 162:1120–1124.e1. doi:10.1016/j.jpeds.2012.11.089. 23324523 PMC3633723

[28] Lahra MM, Beeby PJ, Jeffery HE 2009 Intrauterine inflammation, neonatal sepsis, and chronic lung disease: a 13-year hospital cohort study. Pediatrics 123:1314–1319. doi:10.1542/peds.2008-0656. 19403497

[29] Tröger B, Göpel W, Faust K, Müller T, Jorch G, Felderhoff-Müser U, Gortner L, Heitmann F, Hoehn T, Kribs A, Laux R, Roll C, Emeis M, Mögel M, Siegel J, Vochem M, von der Wense A, Wieg C, Herting E, Härtel C; German Neonatal Network. 2014 Risk for late-onset blood-culture proven sepsis in very-low-birth weight infants born small for gestational age: a large multicenter study from the German Neonatal Network. Pediatr Infect Dis J 33:238–243. doi:10.1097/INF.0000000000000031. 24030351

[30] Miller SE, Maragakis LL 2012 Central line-associated bloodstream infection prevention. Curr Opin Infect Dis 25:412–422. doi:10.1097/QCO.0b013e328355e4da. 22766647

[31] Furuya EY, Dick A, Perencevich EN, Pogorzelska M, Goldmann D, Stone PW 2011 Central line bundle implementation in US intensive care units and impact on bloodstream infections. PLoS One 6:e15452. doi:10.1371/journal.pone.0015452. 21267440 PMC3022589

[32] Strunk T, Power Coombs MR, Currie AJ, Richmond P, Golenbock DT, Stoler-Barak L, Gallington LC, Otto M, Burgner D, Levy O 2010 TLR2 mediates recognition of live Staphylococcus epidermidis and clearance of bacteremia. PLoS One 5:e10111. doi:10.1371/journal.pone.0010111. 20404927 PMC2852418

[33] Chung HM, Cartwright MM, Bortz DM, Jackson TL, Younger JG 2008 Dynamical system analysis of Staphylococcus epidermidis bloodstream infection. Shock 30:518–526. doi:10.1097/SHK.0b013e31816a0b77. 18317411 PMC3677036

[34] Sellman BR, Howell AP, Kelly-Boyd C, Baker SM 2005 Identification of immunogenic and serum binding proteins of Staphylococcus epidermidis. Infect Immun 73:6591–6600. doi:10.1128/IAI.73.10.6591-6600.2005. 16177335 PMC1230897

[35] Sellman BR, Timofeyeva Y, Nanra J, Scott A, Fulginiti JP, Matsuka YV, Baker SM 2008 Expression of Staphylococcus epidermidis SdrG increases following exposure to an in vivo environment. Infect Immun 76:2950–2957. doi:10.1128/IAI.00055-08. 18426874 PMC2446701

[36] Bi D, Qiao L, Bergelson I, Ek CJ, Duan L, Zhang X, Albertsson AM, Pettengill M, Kronforst K, Ninkovic J, Goldmann D, Janzon A, Hagberg H, Wang X, Mallard C, Levy O 2015 Staphylococcus epidermidis bacteremia induces brain injury in neonatal mice via Toll-like receptor 2-dependent and -independent pathways. J Infect Dis 212:1480–1490. doi:10.1093/infdis/jiv231. 25883383 PMC4601917

[37] Kronforst KD, Mancuso CJ, Pettengill M, Ninkovic J, Power Coombs MR, Stevens C, Otto M, Mallard C, Wang X, Goldmann D, Levy O 2012 A neonatal model of intravenous Staphylococcus epidermidis infection in mice <24 h old enables characterization of early innate immune responses. PLoS One 7:e43897. doi:10.1371/journal.pone.0043897. 22970147 PMC3435332

[38] Otto M 2009 Staphylococcus epidermidis—the ‘accidental’ pathogen. Nat Rev Microbiol 7:555–567. doi:10.1038/nrmicro2182. 19609257 PMC2807625

[39] Huyan X-H, Lin Y-P, Gao T, Chen R-Y, Fan Y-M 2011 Immunosuppressive effect of cyclophosphamide on white blood cells and lymphocyte subpopulations from peripheral blood of Balb/c mice. Int Immunopharmacol 11:1293–1297. doi:10.1016/j.intimp.2011.04.011. 21530682

[40] Schaeffer CR, Woods KM, Longo GM, Kiedrowski MR, Paharik AE, Buttner H, Christner M, Boissy RJ, Horswill AR, Rohde H, Fey PD 2015 Accumulation-associated protein enhances Staphylococcus epidermidis biofilm formation under dynamic conditions and is required for infection in a rat catheter model. Infect Immun 83:214–226. doi:10.1128/IAI.02177-14. 25332125 PMC4288872

[41] Natsuka M, Uehara A, Yang S, Echigo S, Takada H 2008 A polymer-type water-soluble peptidoglycan exhibited both Toll-like receptor 2- and NOD2-agonistic activities, resulting in synergistic activation of human monocytic cells. Innate Immun 14:298–308. doi:10.1177/1753425908096518. 18809654

[42] Nilsson M, Frykberg L, Flock JI, Pei L, Lindberg M, Guss B 1998 A fibrinogen-binding protein of Staphylococcus epidermidis. Infect Immun 66:2666–2673. 9596732 10.1128/iai.66.6.2666-2673.1998 PMC108254

